# Concepts and Challenges of Biosimilars in Breast Cancer: The Emergence of Trastuzumab Biosimilars

**DOI:** 10.3390/pharmaceutics10040168

**Published:** 2018-09-25

**Authors:** Alina Uifălean, Maria Ilieş, Raul Nicoară, Lucia Maria Rus, Simona Codruţa Hegheş, Cristina-Adela Iuga

**Affiliations:** 1 Department of Pharmaceutical Analysis, Faculty of Pharmacy, “Iuliu Hațieganu” University of Medicine and Pharmacy, Louis Pasteur Street 6, 400349 Cluj-Napoca, Romania; alina.uifalean@umfcluj.ro (A.U.); ilies.maria@umfcluj.ro (M.I.); raul.nicoara@umfcluj.ro (R.N.); lucia.rus@umfcluj.ro (L.M.R.); iugac@umfcluj.ro (C.-A.I.); 2 Department of Proteomics and Metabolomics, MedFuture Research Center for Advanced Medicine, “Iuliu Hațieganu” University of Medicine and Pharmacy, Louis Pasteur Street 4-6, Gh. Marinescu Street 23, 400349 Cluj-Napoca, Romania

**Keywords:** biosimilars, breast cancer, HER2-positive, trastuzumab, biologic medicines, comparability exercise, interchangeability, substitution

## Abstract

With the development of anti-human epidermal growth factor receptor 2 (HER2) monoclonal antibodies, trastuzumab-based therapy has become the standard of care among patients with early or advanced HER2-positive breast cancer. However, real-world data have shown that up to a half of patients do not receive trastuzumab or any other HER2-targeted agent, mainly due to high treatments costs. The prospect of a more enlarged access to trastuzumab treatment lies in the use of biosimilars, as the European and the US patent of the reference products has or will soon expire. Biosimilars are biologics highly similar in terms of quality characteristics, biological activity, safety and efficacy to already approved biologics. The biosimilarity of any European Union (EU)-approved biosimilar is guaranteed based on the comprehensive comparability exercise which includes comparative analytical, non-clinical and clinical studies. In the matter of biosimilars’ interchangeability and substitution, the European Medicines Agency (EMA) and US Food and Drug Administration (FDA) have adopted different positions, triggering various discussions on the potential immunogenicity and efficacy in individual patients. As more biosimilars are gaining approval, the present review aims to offer concise information for oncologists and pharmacists about the production, approval, interchangeability, and substitution policies of biosimilars used in breast cancer therapy, with a special focus on trastuzumab.

## 1. Introduction

Breast cancer is the leading cause of cancer-related death among females in most parts of the world, including North America, Europe, and Oceania. Despite the increasing incidence patterns, breast cancer mortality has decreased in many high-income states [1]. In the USA, the decline in breast cancer mortality from 1975 to 2010 was mainly attributed to incremental adjustments and advances made in the treatment of breast cancer [2].

One of the major breakthroughs in breast cancer treatment was the development of recombinant humanized monoclonal antibodies (mAbs), such as trastuzumab (anti-human epidermal growth factor receptor (HER2) antibody) and bevacizumab (anti-vascular endothelial growth factor (VEGF) antibody).

Trastuzumab was first approved as a treatment for patients with metastatic HER2-positive breast cancer in 1998 by the US Food and Drug Administration (FDA) and then, in 2000, by the European Medicines Agency (EMA). Later, both authorities approved trastuzumab as adjuvant therapy and EMA also approved trastuzumab as neoadjuvant therapy in early HER2-positive breast cancer patients in 2011 [3,4]. Bevacizumab was approved by EMA in 2006, in combination with paclitaxel or capecitabine for adult patients with metastatic breast cancer [5]. In 2011, FDA revoked the agency’s approval of the breast cancer indication for bevacizumab. Both monoclonal antibodies are included in the World Health Organization (WHO) Model List of Essential Medicines [6]. 

Although trastuzumab-based therapy is considered the standard of care among patients with early or metastatic HER2-positive breast cancer, observational studies on real-world treatment patterns suggest that 12% to 54% of patients across regions of United States, Europe and China did not receive trastuzumab or any other HER2-targeted agent as first and/or later line treatment. The percentages of early breast cancer patients who did not receive (neo)adjuvant trastuzumab are similar. One of the major reasons that impede the access to trastuzumab treatment is drug funding and high treatment costs [7]. 

More accessible treatments could be available in the near future, as biosimilars are emerging, allowing a greater use of anti-HER2 and anti-VEGF therapy. For trastuzumab, the European patent expired in 2014 and the upcoming US patent will expire in 2019. These facts have opened the door for a number of producers to submit applications for trastuzumab biosimilars. Four biosimilars of the reference trastuzumab have already been authorized in Europe and one in the USA. However, the number of trastuzumab biosimilars is expected to grow each year, as other proposed biosimilars with registered phase III clinical trials are awaiting to receive approval from EMA and FDA [7]. 

Facing the forthcoming expansion of monoclonal antibodies biosimilars in breast cancer therapy, health care professionals are questioning how these drugs will integrate into clinical practice. The present review aims to offer concise information about biosimilars used in breast cancer therapy, with a special focus on trastuzumab. Reviewing the latest regulations on their production, approval, interchangeability and substitution policies, we aim to present the scientific principles behind biosimilars, and discuss the most frequent concerns raised by oncologists and pharmacists.

## 2. Biologics, Biosimilars and Intended Copies

As biological medicines are one of the fastest growing sectors in the pharmaceutical industry, it is important for healthcare providers to understand the terminology used for describing these agents. 

Biological medicinal products (or biologics) contain active substances obtained from a biological source, such as living cells or organisms (human, animals, bacteria etc.). Most of the biologics contain proteins, which can differ in size and structural complexity, from simple proteins like insulin to more complex ones such as coagulation factors or monoclonal antibodies. Compared to chemical drugs, which have a well-defined composition, the composition of biologics can be defined only to a certain extent. Moreover, consecutive batches of the same biological medicines can have an inherent degree of minor variability (microheterogenieity). These minor differences must fall within an acceptable range to ensure consistent safety and efficiency [8]. 

The similar biological medicinal products (or biosimilars) are defined by EMA as “products that contain a version of the active substance of an already authorized original biological medicinal product (reference medicinal product)”, being similar “in terms of quality characteristics, biological activity, safety and efficacy based on a comprehensive comparability exercise”. It is important to understand that biosimilars are not generic forms of a reference product. Due to the natural variability and more complex manufacturing of biologics, the generic approach which involves demonstration of bioequivalence with a reference medicine, applicable to most chemically-derived medicines, is not sufficient to demonstrate the similarity of biological-derived products. In addition to comparative pharmacokinetic and pharmacodynamic studies, safety and efficacy data are usually required to establish biosimilarity with the reference product [8,9]. 

Similarly, the FDA defines biosimilars as “biological products that are highly similar to and have no clinically meaningful differences from an existing FDA-approved reference product in terms of safety, purity, and potency (safety and effectiveness)”. Furthermore, the FDA has defined a particular category of biosimilars, the interchangeable biosimilars products. In addition to being biosimilars, these products meet additional requirements to ensure they deliver the same clinical result as the reference product in any given patient [10]. Aspects of interchangeability will be discussed later. 

All the biosimilars approved by FDA, EMA or WHO undergo a rigorous evaluation to ensure the efficacy, safety, and quality of these products. In contrast, intended copies (or non-comparable biotherapeutics) of biological products are copies of a reference medicine which have not undergone the stringent comparability evaluations and authorization procedures required by the major regulatory agencies. Although intended copies are being commercialized in some countries, evidence of rigorous clinical studies is incomplete or lacking and their quality profiles are only partially known. Therefore, differences in formulation, dosages, efficacy or safety cannot be excluded. It is important that oncologists distinguish between biosimilars and intended copies in order to ensure safe treatments for their patients [11].

## 3. Obtaining Biologics (and Biosimilars)

The characteristics of the final biological product are highly dependent on the manufacturing process. The manufacturing information of the reference medicine is private and, since it is not all publicly available, biosimilar manufacturers must develop an entirely new customized process [12]. Even small changes in manufacturing can result in altered protein stability, including different post-translational modifications such as glycosylation. These changes may influence the biologic activity, as well as the overall efficacy, safety and immunogenicity [13,14].

Biologics are often produced by modern genetic engineering techniques. In particular, mAbs, depending on the degree of “humanization”, are generated by recombinant DNA (rDNA) technology, hybridoma technology, B lymphocyte immortalization or other technologies (e.g., display technology, genetically engineered animals) [15]. 

The first mAbs were obtained using hybridoma technology, by fusion of B-lymphocytes from immunized mice or rats with murine myeloma cells. Despite the great success of the technology, the clinical use of the resulting molecules was hampered by the development of human anti-murine antibody (HAMA) response in most treated patients. The HAMA response can trigger important immunogenic reactions and impair the treatment efficacy due to fast clearance of the murine monoclonal antibodies [16]. 

To diminish the HAMA response, humanized antibodies, such as trastuzumab and bevacizumab, were later developed. The protein sequence of a humanized antibody is essentially identical to that of a human variant, despite the non-human origin of some of its complementarity determining region (CDR) segments responsible for the antibody’s ability to bind to its target antigen. Even if the humanization of antibodies can be accomplished through a number of techniques, the most important one is based on the insertion of relevant CDRs into a human antibody framework (CDR grafting), when the resulting “humanized” antibodies contain 85–90% human sequences. Functionally, the process involves the insertion of the appropriate CDR coding segments (responsible for the desired binding properties) into a human antibody structure. To achieve this, recombinant DNA methods are used, leading to the use of an appropriate vector with subsequent expression in mammalian cells. After the antibody with the desired properties is developed to be produced in murine or other non-human species, the coding DNA of the mAb will be isolated, cloned into a vector, and then sequenced. The DNA sequence of the desired CDRs will be determined and inserted into a construct containing the DNA for a human antibody variant [17,18].

Most of the therapeutic mAbs available today are genetic engineered. They can have the advantages of decreased immunogenicity, enhanced in vivo circulating half-life, but they still can stimulate the human immune system to produce the HAMA response. Moreover, for biosimilars there are additional challenges due to the structural/conformational changes determined by the new manufacturing conditions. To ensure that these changes do not alter the safety and the efficacy of the final product, EMA and FDA guidelines on biosimilars approval require that each biosimilar must go through a thorough comparability exercise with the reference product, as detailed in Section 5 [13,14,15,19].

## 4. Biosimilars Used in Breast Cancer Therapy

The biological agents that are currently being used in breast cancer clinical routine include hematopoietic growth factors and mAbs. Biosimilars of growth factors such as erythropoietin and filgrastim have already entered the market and are widely used in the management of breast cancer therapy. EMA approved the first biosimilar of filgrastim in 2008. It was not until November 2017 when the first biosimilar of the monoclonal antibody trastuzumab had received European regulatory approval for the treatment of early breast cancer, metastatic breast cancer, and metastatic gastric cancer. The currently EMA and FDA-approved biosimilars indicated in breast cancer treatment, adjunct treatment or prevention of treatment related side-effects are presented in Table 1.

Besides these already approved biosimilars, there are several proposed biosimilars of trastuzumab with registered phase III clinical trials for early or metastatic breast cancer which are currently ongoing and might soon become available [7].

## 5. Biosimilar Comparability Exercise

The EU-approval of a biosimilar implies extensive comparison tests between the submitted biosimilar and the reference medicine in order to demonstrate overall biosimilarity. These “comparability studies” represent the cornerstone of a biosimilar development and involve head-to-head comparison tests designed to investigate whether there are clinically meaningful differences between the biosimilar and the reference medicine in terms of efficacy, safety, and potency. 

**Figure 1:**
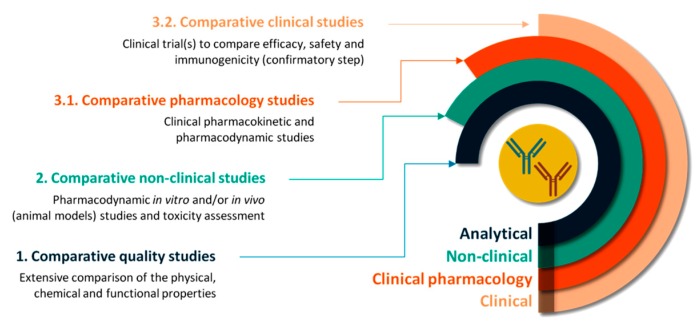
The step-wise process of the comparability exercise required to demonstrate the biosimilarity of a trastuzumab biosimilar to its reference product.

The entire process is organized in a three-step manner, comprising comparability quality studies, comparative non-clinical studies and comparative clinical (pharmacology) studies (Figure 1). The extent and the nature of data required in each step depend on the results obtained in the previous steps so that any differences in clinical efficiency are precluded [8]. Similarly, FDA recommends producers a stepwise approach to developing the data and information needed to support the demonstration of biosimilarity [20].

### 5.1. Comparative Quality Studies

The comparative quality studies involve demonstration of the physicochemical similarity between the biosimilar and the reference medicine along with biological activity comparison. In fact, compared to the approval procedure of the reference medicine, most of the emphasis in the biosimilar approval is placed on extensive structural and functional characterization. The methods used in this step include accurate and sensitive analytical techniques able to detect minor differences between the tested medicines. Any difference found in this step must be further investigated as it may affect the final efficacy and safety profile [8,21]. As an example, Table 2 presents some of the comparative quality tests needed to demonstrate biosimilarity between the biosimilar versions of trastuzumab and its reference medicine, Herceptin^®^ (Roche Registration GmbH) [22,23,24].

### 5.2. Comparative Non-Clinical Studies

The comparative non-clinical studies aim to compare the pharmacodynamic and the toxic properties of the two tested medicines. As mentioned, the type and the extent of non-clinical in vitro tests depend on the level of evidence acquired in the previous step. Pharmacodynamic in vivo experiments using animal models are required only in the absence of suitable in vitro models [8].

### 5.3. Comparative Clinical Studies

The last step, the comparative clinical studies are tailored to investigate the dissimilarities observed in the physicochemical, biological or in vitro properties and to address any uncertainties from previous steps. The final purpose is to rule out any clinically relevant differences between the biosimilar and the reference and to confirm biosimilarity. As the reference medicine has already proved efficacy and safety in patients, the biosimilar will need to demonstrate comparable efficacy and safety profiles to those of the reference medicine. Usually, the clinical studies evaluate the pharmacokinetic/pharmacodynamic profiles of the biosimilar and reference and the immunogenicity, safety, and efficacy profiles [8,25].

Unlike less complex biologics, which have sensitive pharmacodynamic markers used as surrogate markers of clinical efficacy (i.e., the hemoglobin levels for the erythropoietin treatment), current mAbs have no such surrogate markers. Therefore, to demonstrate comparability, cancer mAbs must rely on clinical endpoints such as progression-free/disease-free survival (PFS/DFS) or overall survival (OS) [26,27].

The clinical efficacy and safety of trastuzumab biosimilars already approved in Europe were assessed in moderately sized, randomized, double-blind phase III studies. Generally, the primary efficacy endpoint set was the rate of patients achieving pathological complete response (pCR), defined as the absence of invasive tumor cells in the breast and in axillary lymph nodes regardless of ductal carcinoma in situ (DCIS). The secondary endpoints evaluated were either the pCR in breast tissue only, the pCR in breast and axilla tissue in the absence of DCIS, the overall response rate, the PFS, the DFS, the OS or the event-free survival [22,23,24]. The treatment groups, interventions, primary-endpoints and outcomes of the clinical trials conducted for the trastuzumab biosimilars approved in Europe so far are compared in Table 3.

For each trastuzumab biosimilar authorized in Europe, data from the comparative quality, the non-clinical and the clinical studies have supported biosimilarity with the reference medicine. Moreover, as the mechanism of action of trastuzumab is similar in different conditions such as early and metastatic HER2-positive breast cancer, HER2-positive gastric cancer and based on the overall comparability exercise, extrapolation to the other indications in oncology was accepted [22,23,24].

## 6. Interchangeability, Switching and Substitution

### 6.1. Definitions 

The main advantage offered by biosimilars is the enlarged patient access to biological medicines with proven pharmaceutical quality. However, in the context of an emerging biosimilar market, oncologists might come up against the situation of choosing between the reference drug and a biosimilar or between biosimilars. In this light, it is important for specialists to differentiate between interchangeability, switching and substitution.

According to EMA, interchangeability refers to the possibility of replacing a reference medicine with a biosimilar or one biosimilar to another, with the intention of obtaining the same clinical effect. When the decision about replacement is taken by the prescriber, the exchange is done by switching. When the decision of dispensing one medicine instead of another equivalent and interchangeable medicine is taken by the pharmacist, without consulting the prescriber, the replacement is referred as (automatic) substitution [8]. The major concerns about switching from reference medicines to biosimilars include enhanced immunogenicity, compromised safety, diminished efficacy, all leading to altered clinical outcomes.

### 6.2. Food and Drug Administration (FDA) and European Medicines Agency (EMA) Positions

The two major regulatory authorities, the EMA and FDA, have adopted different positions on defining the biosimilars interchangeability.

According to the FDA, the approval of a biosimilar does not imply an automatic interchange with the reference medicine or with another biosimilar. In order for a biosimilar to be labeled as interchangeable, the agency requires additional evidence to demonstrate that the biosimilar is interchangeable, i.e., “can be expected to produce the same clinical result as the reference product in any given patient”. Moreover, “for a biological product that is administered more than once to an individual, the risk in terms of safety or diminished efficacy of alternating or switching between use of the biological product and the reference product is not greater than the risk of using the reference product without such alternation or switch” [29]. In other words, an interchangeable biosimilar could be use by any patient, at any given time, guaranteeing the same efficacy and safety as the reference medicine.

In January 2017, the FDA has released a draft guidance for industries containing detailed consideration in demonstrating interchangeability with a reference product. The guide proposes a dedicated switching study design, with a lead-in period of treatment with the reference product, followed by a randomized two-arm period—with one arm incorporating switching between the proposed interchangeable product and the reference product (switching arm) and the other remaining as a non-switching arm receiving only the reference product [29].

In the US, only the biosimilars approved as interchangeable can be automatically substituted for their reference product at the pharmacy level. However, the automatic substitution in the US is subjected to individual state law. As no interchangeable biosimilar has been approved so far, any switch of the reference medicine with a biosimilar or between biosimilars must require the prescribing doctor’s consent.

On the other hand, the EMA has abstained from releasing any recommendation on interchangeability and automatic substitution of biosimilars with the reference medicine, leaving the decision to the responsibility of the member states. According to the EMA, any switching should be consent by the prescriber in consultation with the patient, taking into account the patient’s medical history, as well as the local prescribing regulations [8]. As a result, most European countries have endorsed switching under the supervision of the prescriber, while disallowing automatic substitution.

### 6.3. Challenges and Current Knowledge 

The different positions of the FDA and EMA, along with the prudent position adopted by the EMA, have raised several questions about biosimilars’ interchangeability. 

On the one hand, the extensive comparability tests applied to confirm biosimilarity are designed to identify clinically meaningful differences between the reference product and the proposed biosimilar in terms of safety, purity, and potency. Therefore, it is expected that two medicines which have the same mechanism of action, similar physicochemical properties, similar pharmacokinetic and pharmacodynamic profiles and comparable safety and efficacy to induce the same clinical outcome in a single patient. On the other hand, biosimilars are produced in different cell lines or bacteria, starting from a DNA sequence which will encode a protein subjected to various post-translational changes. These unknown changes in folding or post-translational structure have raised potential concerns related to immunogenicity. The safety and efficacy of biosimilars are rigorously tested through the comparability exercise, but still require continuous monitoring once the biosimilar is approved [30].

Moreover, there are limited data about the consequences of switching between a reference product and its biosimilars. As the number of biosimilars is continuously growing, patients may switch between two or more biosimilars whose interchangeability has not been assessed. The importance of additional data to support interchangeability has been issued in draft guidance on interchangeability emitted by the FDA [29].

On the other hand, specific interchangeability studies could be very complex and time-consuming. Besides, the experience accumulated in the past decade and the results of the comparative studies completed so far have shown no evidence of safety concerns [31,32,33].

A recent study has compared safety, tolerability, and immunogenicity, after switching the treatment from trastuzumab to a proposed biosimilar in early HER2-positive breast cancers (NCT number: 01901146) [33,34]. Previously untreated female patients with early HER2-positive breast cancers (*n* = 827) who have completed four cycles of run-in anthracycline-based chemotherapy were assigned 1:1 to receive paclitaxel plus either the biosimilar ABP 980 or the reference trastuzumab in the neoadjuvant phase. After surgical resection of the breast tumor, patients were randomly assigned at baseline to continue APB 980, continue trastuzumab, or switch from trastuzumab to APB 980, as adjuvant treatment. The results showed that safety and immunogenicity were similar in patients who were switched and in those who continued to receive trastuzumab and both groups had similar safety outcomes in both neoadjuvant and adjuvant phases [34].

Another review of data related to switching between human recombinant growth hormones, erythropoietins and granulocyte colony stimulating agents found no evidence from clinical trial data or post-marketing surveillance data that switching to and from different biopharmaceuticals leads to safety concerns [31].

A general analysis of the theoretical grounds of potential switch-related adverse effects concluded that extensive demonstration of biosimilarity, combined with intensified post-marketing surveillance is a sufficient and realistic way to ensure interchangeability of EU-approved biosimilars under the supervision of the prescriber [32].

## 7. Substitution Policies across Europe

In most EU countries, the decision to treat a patient with a biosimilar or with the reference medicine is left to the clinical assessment of the physician. In Finland, the EU biosimilars are considered interchangeable with their reference products under the supervision of a health care worker and any switch should be documented [35]. In the Netherlands, any exchange between biological medicines is permitted, but only if the patient is accurately informed and clinically supervised [36]. In Ireland, the Health Products Regulatory Authority (HPRA) does not recommend that patients are switched back and forth between the reference product and a biosimilar and prescribers, pharmacists and procurement staff should discuss to decide the treatment options [37]. In Romania, legislation provides that prescribers must use the brand name as well as the international non-proprietary name (INN) when prescribing biologics. The pharmacists must dispense exactly the biological product prescribed by the physician, any automatic substitution of biologics being forbidden [38].

France is a strong supporter of biosimilars and the first European country to explicitly permit biosimilar substitution. According to the National Health Strategy, France intends to reach the goal of 80% biosimilars penetration by 2022 [39]. Pharmacists in France are allowed to substitute a biosimilar for the prescribed (reference) biological either at treatment initiation or to ensure continuity with the same biosimilar, if the biosimilar belongs to the same “similar biologic group” as the prescribed product. The replacement is allowed when the prescribing physician has not explicitly prohibited it by marking the prescription as “non-substitutable”. However, substitution is not yet implemented in practice as no decree has been enacted so far [40,41]. In Germany, substitution is possible for specific groups of biosimilars (e.g., produced by the same manufacturer and through the same manufacturing process), unless specifically forbidden by the prescriber. Similarly, in Estonia, Latvia, Poland, and Russia, substitution of biologics is allowed under certain regulations [40].

## 8. Conclusions

The high costs of anticancer mAbs have become a burden on public health care systems not only in low-income countries but also in developed countries. In an effort to lower costs and increase access to treatment, the WHO has launched a pilot program inviting manufacturers to submit applications for prequalification of trastuzumab biosimilars into their List of Essential Medicines [42]. The cost saving generated with the introduction of trastuzumab biosimilars is estimated to be 20–30% lower compared to the cost of the reference product [7]. Even with a discount as low as 15%, the cost savings from the introduction of biosimilar trastuzumab would reach €0.26 million in the first year, as calculated in Croatia [43].

The extent to which biosimilars will contribute to the reduction of health care budgets and will be integrated into the management of cancer treatment depends on their level of acceptance by regulators, prescribers, pharmacists and patients [44].

To date, no clinically meaningful differences in safety, efficacy, and immunogenicity have been reported after switching a reference product to its biosimilar version. In the near future, the accumulated clinical experience will straighten out any persisting uncertainties related to biosimilars’ use. Meanwhile, the replacement of the reference medicine with a biosimilar should involve careful patient monitoring under the supervision of the prescriber, active adverse event reporting, and vigilant post-marketing surveillance.

## Figures and Tables

**Table 1:** Biosimilars approved by the European Medicines Agency (EMA) and the US Food and Drug Administration (FDA) which can be indicated in breast cancer therapy (August 2018).

Active Substance	Reference Trade Name/Manufacturer	Biosimilar Trade Name/Manufacturer	Authorization Date
**EMA**			
**epoetin alpha**	Eprex/Erypo/Janssen-Cilag Pharma GmbH	Abseamed/Medice Arzneimittel Pütter GmbH & Co. KG	08/2007
		Binocrit/Sandoz GmbH	08/2007
		Epoetin Alfa Hexal/Hexal AG	08/2007
**epoetin zeta**	Eprex/Erypo/Janssen-Cilag Pharma GmbH	Retacrit/Hospira UK Limited	12/2007
		Silapo/Stada Arzneimittel AG	12/2007
**filgrastim**	Neupogen/Amgen Europe B.V.	Tavagrastim/Teva GmbH	09/2008
		Ratiograstim/Ratiopharm GmbH	09/2008
		Zarzio/Sandoz GmbH	02/2009
		Filgrastim Hexal/Hexal AG	02/2009
		Nivestim/Hospira UK Ltd.	06/2010
		Grastofil/Apotex Europe BV	10/2013
		Accofil/Accord Healthcare Ltd.	09/2014
**bevacizumab**	Avastin/Roche Registration GmbH	Mvasi/Amgen Europe B.V.	01/2018
**trastuzumab**	Herceptin/Roche Registration GmbH	Ontruzant/Samsung Bioepis UK Limited (SBUK)	11/2017
		Herzuma/Celltrion Healthcare Hungary Kft.	02/2018
		Kanjinti/Amgen Europe B.V.	05/2018
		Trazimera/Pfizer Europe MA EEIG	07/2018
**FDA**			
**epoetin alpha-epbx**	Epogen/Procrit/Amgen Inc	Retacrit/Hospira INC	05/2018
**Filgrastim-sndz**	Neupogen/Amgen Inc.	Zarxio/Sandoz GmbH	03/2015
		Nivestym (filgrastim-aafi)/Pfizer	07/2018
**Pegfilgrastim-jmdb**	Neulasta/Amgen Inc	Fulphila/Mylan GmbH	06/2018
**Trastuzumab-dkst**	Herceptin/Genentech Inc	Ogivri/Mylan GmbH	12/2017

**Table 2:** Examples of comparable quality studies performed to demonstrate biosimilarity between the European Union (EU)-approved trastuzumab biosimilars and the reference medicine [22,23,24].

Purpose	Features/Parameters	Proposed Analytical Methods
Primary structure analysis	- molar extinction coefficient - level of free thiol groups - intact mass analysis - N-terminal and C-terminal sequencing - reduced and non-reduced peptide mapping (assessment of deamidation, oxidation and isomerisation) - disulphide bonds - glican mapping (% of afucosylation, galactosylation, sialylation, etc.) - N-linked glycan - isoelectric point	- UV spectrometry - RP-HPLC - peptide mapping by LC-MS - CIF
Higher order structure analysis	- protein structure - protein conformation - protein folding and stability (melting temperature)	- FTIR - far-UV CD - near-UV CD - H/DX - DSC
Particles and aggregates	- particle flow, number, velocity, size - sub-visible particles - sedimentation coefficients	- MFI; - AUC-SV; - HIAC; - FFF;
Purity and impurities	- levels of monomers, high molecular weights, low molecular weights - levels of heavy and light chain - residual impurities	- SE-HPLC-MALLS - Reduced CE-SDS - Residual Host Cell Protein - Residual Host Cell DNA - Residual Protein A
Content	- protein concentration	- UV spectrometry (A_280_)
Biological activity	- cell inhibition	- in vitro antiproliferation assays
	- ADCC activity	- ADCC assay
	- Binding affinity—for HER2, for Fc receptors (FcγRIIIa V, FcγRIIIa F, FcγRIIIb, FcγRIIa, FcγRIIb, FcγRI, FcRn), for C1q	- ELISA - Surface plasmon resonance assay

ultraviolet spectrometry (UV); reverse-phase high performance liquid chromatography (RP-HPLC); liquid chromatography—mass spectrometry (LC-MS); capillary isoelectric focusing (CIF); Fourier-transform infrared spectroscopy (FTIR); far-UV circular dichroism (far-UV CD); near-UV circular dichroism (near-UV CD); hydrogen/deuterium exchange (H/DX); differential scanning calorimetry (DSC); micro flow imaging (MFI); analytical ultracentrifugation sedimentation velocity (AUC-SV); high accuracy light obscuration particle counting (HIAC); field flow fractionation (FFF); size exclusion high performance liquid chromatography coupled with multiangle laser light scattering with light scattering detection (SE-HPLC-MALLS); capillary electrophoresis—sodium dodecyl sulphate (CE-SDS); antibody-dependent cell-mediated cytotoxicity (ADCC); enzyme-linked immunosorbent assay (ELISA).

**Table 3:** Treatment groups, interventions and efficacy parameters of the clinical trials conducted for the EU-approval of trastuzumab biosimilars.

Trastuzumab Biosimilar	Study Identifier	Treatment Groups	Intervention	Primary End-Point	Primary Endpoint Outcomes		Ref.
Ontruzant^®^ (Samsung Bioepis UK Limited)	SB3-G31-BC	- SB3 (*n* = 437) - Herceptin^®^ (*n* = 438)	- 8 mg/kg loading followed by 6 mg/kg every 3 weeks for a total of 8 neoadjuvant and 10 adjuvant cycles, given IV - neoadjuvant docetaxel, followed by 5-fluorouracil, epirubicin and cyclophosphamide	No histological evidence of residual invasive tumour cells in the breast specimen removed at surgery	SB3	Herceptin^®^	[22]
					PPS 51.7% FAS 49.0%	PPS 42.0% FAS 39.7%	
Herzuma^®^ (Celltrion Healthcare Hungary Kft.)	CT-P6 3.2	- CT-P6 (*n* = 271) - Herceptin^®^ (*n* = 278)	- 8 mg/kg (cycle 1), 6 mg/kg (cycles 2 through 8), given IV	pCR (absence of invasive tumour cells in the breast and in axillary lymph nodes, regardless of the DCIS)	CT-P6	Herceptin^®^	[23]
					PPS 46.8%	PPS 50.4%	
Kanjinti^®^ (Amgen Europe B.V.)	20120283	- ABP980 (as neoadjuvant *n* = 364, as adjuvant *n* = 349) - Herceptin^®^ (as neoadjuvant *n* = 361, as adjuvant *n* = 171)	Neoadjuvant: Tested drug + Paclitaxel Tested drug: initial dose 8 mg/kg, subsequent 6 mg/kg every 3 weeks for 3 cycles, given IV	pCR (pathologic complete response in breast and axillary tissue regardless of DCIS)	ABP980	Herceptin^®^	[24]
					48.0% regardless of DCIS	40.5% regardless of DCIS	
			Adjuvant: - ABP980 6 mg/kg IV every 3 weeks up to 1 year - Herceptin^®^ 6 mg/kg IV every 3 weeks up to 1 year OR ABP980 6 mg/kg IV every 3 weeks up to 1 year				
Trazimera^®^ (Pfizer Europe MA EEIG)	B3271002	- PF-05280014 (*n* = 352) - Herceptin^®^ (*n* = 355)	- 4 mg/kg (loading dose), 2 mg/kg (weekly until week 33), given IV	ORR (CR or PR by week 25 [±14 days] and confirmed on a follow-up assessment	PF-05280014	Herceptin^®^	[28]
					ORR 62.5%	ORR 66.5%	

intravenously (IV); per-protocol set (PPS); full analysis set (FAS); pathologic complete response (pCR); ductal carcinoma in situ (DCIS); complete response (CR); partial response (PR); objective response rate (ORR).

## References

[1] Torre L.A., Siegel R.L., Ward E.M., Jemal A. (2016). Global cancer incidence and mortality rates and trends—An update. Cancer Epidemiol. Biomarkers Prev..

[2] Narod S.A., Iqbal J., Miller A.B. (2015). Why have breast cancer mortality rates declined?. J. Cancer Policy.

[3] US FDA Full Prescribing Information for Herceptin (Trastuzumab). https://www.accessdata.fda.gov/drugsatfda_docs/label/2010/103792s5250lbl.pdf.

[4] EMA European Public Assessment Report of Herceptin (Trastuzumab). http://www.ema.europa.eu/docs/en_GB/document_library/EPAR_-_Product_Information/human/000278/WC500074922.pdf.

[5] EMA European Public Assessment Report of Avastin (Bevacizumab). http://www.ema.europa.eu/docs/en_GB/document_library/EPAR_-_Summary_for_the_public/human/000582/WC500029260.pdf.

[6] WHO Model List of Essential Medicines—20th List. http://www.who.int/medicines/publications/essentialmedicines/en/.

[7] Blackwell K., Gligorov J., Jacobs I., Twelves C. (2018). The global need for a trastuzumab biosimilar for patients with HER2-positive breast cancer. Clin. Breast Cancer.

[8] EMA and EU Biosimilars in the EU. Information Guide for Healthcare Professionals. http://www.ema.europa.eu/docs/en_GB/document_library/Leaflet/2017/05/WC500226648.pdf.

[9] EMA Guideline on Similar Biological Medicinal Products. http://www.ema.europa.eu/docs/en_GB/document_library/Scientific_guideline/2014/10/WC500176768.pdf.

[10] US FDA Biological Product Definitions. https://www.fda.gov/downloads/Drugs/DevelopmentApprovalProcess/HowDrugsareDevelopedandApproved/ApprovalApplications/TherapeuticBiologicApplications/Biosimilars/UCM581282.pdf.

[11] Jacobs I., Ewesuedo R., Lula S., Zacharchuk C. (2017). Biosimilars for the Treatment of Cancer: A systematic Review of Published Evidence. BioDrugs.

[12] Lee J.F., Litten J.B., Grampp G. (2012). Comparability and Biosimilarity: Considerations for the Healthcare Provider. Curr. Med. Res. Opin..

[13] US FDA Quality Considerations in Demonstrating Biosimilarity of a Therapeutic Protein Product to a Reference Product. Guidance for Industry. https://www.fda.gov/downloads/drugs/guidances/ucm291134.pdf.

[14] Laslop A. (2013). Biosimilar Monoclonal Antibodies—Challenges and Opportunities in Europe. GaBI J..

[15] EMA Guideline on Development, Production, Characterization and Specification for Monoclonal Antibodies and Related Products. http://www.ema.europa.eu/docs/en_GB/document_library/Scientific_guideline/2016/08/WC500211640.pdf.

[16] Tjandra J.J., Ramadi L., McKenzie I.F. (1990). Development of human anti-murine antibody (HAMA) response in patients. Immunol. Cell Biol..

[17] Glassy M.C., Gupta R. (2014). Technical and ethical limitations in making human monoclonal antibodies (an overview). Methods Mol. Biol..

[18] EMA Production and Quality Control of Monoclonal Antibodies. http://www.ema.europa.eu/docs/en_GB/document_library/Scientific_guideline/2009/09/WC500003444.pdf.

[19] Goncalves J., Araujo F., Cutolo M., Fonseca J.E. (2016). Biosimilar monoclonal antibodies: Preclinical and clinical development aspects. Clin. Exp. Rheumatol..

[20] US FDA Scientific Considerations in Demonstrating Biosimilarity to a Reference Product. Guidance for Industry. https://www.fda.gov/downloads/Drugs/GuidanceComplianceRegulatoryInformation/Guidances/UCM291128.pdf.

[21] Rugo H.S., Linton K.M., Cervi P., Rosenberg J.A., Jacobs I. (2016). A clinician’s guide to biosimilars in oncology. Cancer Treat. Rev..

[22] EMA Assessment Report for Ontruzant, Procedure no. EMEA/H/C/004323/0000. http://www.ema.europa.eu/docs/en_GB/document_library/EPAR_-_Public_assessment_report/human/004323/WC500242488.pdf.

[23] EMA Assessment Report for Herzuma, Procedure no. EMEA/H/C/002575/0000. http://www.ema.europa.eu/docs/en_GB/document_library/EPAR_-_Public_assessment_report/human/002575/WC500249108.pdf.

[24] EMA Assessment Report for Kanjinti, Procedure no. EMEA/H/C/004361/0000. http://www.ema.europa.eu/docs/en_GB/document_library/EPAR_-_Public_assessment_report/human/004361/WC500249709.pdf.

[25] Pasina L., Casadei G., Nobili A. (2016). Biological agents and biosimilars: Essential information for the internist. Eur. J. Intern. Med..

[26] Thill M. (2015). New frontiers in oncology: Biosimilar monoclonal antibodies for the treatment of breast cancer. Expert Rev. Anticancer Ther..

[27] EMA Guideline on Similar Biological Medicinal Products Containing Monoclonal Antibodies—Non-Clinical and Clinical Issues. http://www.ema.europa.eu/docs/en_GB/document_library/Scientific_guideline/2012/06/WC500128686.pdf.

[28] EMA Assessment Report for Trazimera, Procedure No. EMEA/H/C/004463/0000. http://www.ema.europa.eu/docs/en_GB/document_library/EPAR_-_Public_assessment_report/human/004463/WC500254124.pdf.

[29] US FDA Considerations in Demonstrating Interchangeability with a Reference Product. Guidance for Industry—Draft. https://www.fda.gov/downloads/Drugs/GuidanceComplianceRegulatoryInformation/Guidances/UCM537135.pdf.

[30] Patel D., Gillis C., Naggar J., Mistry A., Mantzoros C.S. (2017). The rise of biosimilars: How they got here and where they are going. Metabolism.

[31] Ebbers H.C., Muenzberg M., Schellekens H. (2012). The safety of switching between therapeutic proteins. Expert Opin. Biol. Ther..

[32] Kurki P., van Aerts L., Wolff-Holz E., Giezen T., Skibeli V., Weise M. (2017). Interchangeability of biosimilars: A European perspective. BioDrugs.

[33] Declerck P., Bakalos G., Zintzaras E., Barton B., Schreitmuller T. (2018). Monoclonal antibody biosimilars in oncology: Critical appraisal of available data on switching. Clin. Ther..

[34] Von Minckwitz G., Colleoni M., Kolberg H.C., Morales S., Santi P., Tomasevic Z., Zhang N., Hanes V. (2018). Efficacy and safety of ABP 980 compared with reference trastuzumab in women with HER2-positive early breast cancer (LILAC study): A randomised, double-blind, phase 3 trial. Lancet Oncol..

[35] FIMEA Interchangeability of Biosimilars—Position of Finnish Medicines Agency. https://www.fimea.fi/documents/542809/838272/29197_Biosimilaarien_vaihtokelpoisuus_EN.pdf.

[36] MEB Are Biosimilar Medicines Interchangeable?—Position of Medicines Evaluation Board. https://english.cbg-meb.nl/human/healthcare-providers/biosimilar-medicines.

[37] HPRA Guide to Biosimilars for Healthcare Professionals and Patients—Position of Health Products Regulatory Authority. https://www.hpra.ie/docs/default-source/publications-forms/guidance-documents/guide-to-biosimilars-for-healthcare-professionals-and-patients-v2.pdf?sfvrsn=18.

[38] Monitorul Oficial al României, no. 270 of March 27, 2018—Decision No. 140/2018 of March 21, 2018. http://www.monitoruloficial.ro/article--e-Monitor--339.html.

[39] Le Ministère des Solidarités et de la Santé—Stratégie Nationale de Santé 2018–2022. http://solidarites-sante.gouv.fr/IMG/pdf/dossier_sns_2017_vdefpost-consult.pdf.

[40] Moorkens E., Vulto A.G., Huys I., Dylst P., Godman B., Keuerleber S., Claus B., Dimitrova M., Petrova G., Sovic-Brkicic L. (2017). Policies for biosimilar uptake in Europe: An overview. PLoS ONE.

[41] Generics and Biosimilars Initiative (GaBI online) France to Allow Biosimilars Substitution. http://www.gabionline.net/Policies-Legislation/France-to-allow-biosimilars-substitution.

[42] WHO Pilot Procedure for Prequalification of Biotherapeutic Products: Rituximab and Trastuzumab. http://www.who.int/medicines/regulation/prequalification/01_Pilot_Prequalification_BTPs_June2018.pdf.

[43] Cesarec A., Likic R. (2017). Budget impact analysis of biosimilar trastuzumab for the treatment of breast cancer in Croatia. Appl. Health Econ. Health Policy.

[44] Cazap E., Jacobs I., McBride A., Popovian R., Sikora K. (2018). Global acceptance of biosimilars: Importance of regulatory consistency, education, and trust. Oncologist.

